# Genome-Wide Identification of SNAC1-Targeted Genes Involved in Drought Response in Rice

**DOI:** 10.3389/fpls.2019.00982

**Published:** 2019-07-26

**Authors:** Xu Li, Yu Chang, Siqi Ma, Jianqiang Shen, Honghong Hu, Lizhong Xiong

**Affiliations:** ^1^National Key Laboratory of Crop Genetic Improvement, National Center of Plant Gene Research (Wuhan), Huazhong Agricultural University, Wuhan, China; ^2^Department of Biology, University of Pennsylvania, Philadelphia, PA, United States

**Keywords:** SNAC1, stress response, transcriptional regulation, ChIP-Seq, RNA-Seq

## Abstract

Drought stress can cause huge crop production losses. Drought resistance consists of complex traits, and is regulated by arrays of unclear networks at the molecular level. A stress-responsive NAC transcription factor gene *SNAC1* has been reported for its function in the positive regulation of drought resistance in rice, and several downstream SNAC1 targets have been identified. However, a complete regulatory network mediated by SNAC1 in drought response remains unknown. In this study, we performed Chromatin immunoprecipitation sequencing (ChIP-Seq) and RNA-Seq of *SNAC1*-overexpression transgenic rice (*SNAC1*-OE) lines and wild-type under normal and moderate drought stress conditions, to identify all SNAC1 target genes at a genome-wide scale by RNA-Seq analyses. We detected 980 differentially expressed genes (DEGs) in the *SNAC1*-OE lines compared to the wild-type control under drought stress conditions. By ChIP-Seq analyses, we identified 4,339 SNAC1-binding genes under drought stress conditions (SNAC1BGDs). By combining the DEGs and SNAC1BGDs, we identified 93 SNAC1-targeted genes involved in drought responses (SNAC1TGDs). Most SNAC1TGDs are involved in transcriptional regulation, response to water loss, and other processes related to stress responses. Moreover, the major motifs in the SNAC1BGDs promoters include a NAC recognition sequence (NACRS) and an ABA responsive element (ABRE). *SNAC1*-OE lines are more sensitive to ABA than wild-type. SNAC1 can bind to the *OsbZIP23* promoter, an important ABA signaling regulator, and positively regulate the expression of several ABA signaling genes.

## Introduction

Sessile plants unavoidably encounter diverse environmental stresses during their growth and development. Among the adverse stresses, water deficit, or drought can cause huge yield loss or even loss of the entire crop harvest. It has been a long-lasting goal to completely unveil the mechanisms of drought response and to develop new tools for drought resistance improvement in stable food crops such as rice (Hu and Xiong, 2014), since irrigated rice, which is more sensitive to water deficit than upland crops, frequently suffers from drought stress in the rice planting regions worldwide (Hussain and Mumtaz, 2014).

Plants respond to drought stress through a series of known (such as abscisic acid [ABA]-dependent) and unknown signaling pathways, which ultimately lead to morphological, physiological, and biochemical changes to adapt to the drought conditions (Xiong et al., 2002). Transduction of the drought stress signal activates diverse regulatory proteins and modulates expression changes of numerous stress-responsive genes to enable plant survival. Among the regulatory proteins, many transcription factors (TFs) belonging to diverse families play important roles in drought resistance, through directly or indirectly regulating the expression of downstream genes under drought stress conditions, through feedback regulation of the upstream stress signaling, and/or interacting with other regulatory proteins to form a complex network (Xiong et al., 2002; Hirayama and Shinozaki, 2010; Santos et al., 2011; Song et al., 2016; Samad et al., 2017).

NAC (NAM, ATAF1/2, and CUC2) transcription factors comprise one of the largest TF families, which are found only in plants (Sun et al., 2015). NAC proteins are identified by a highly conserved DNA binding domain which is termed as the NAC domain in the N-terminal region, whereas the transcription regulatory region in the C-terminal domain in NAC proteins is usually diversified both in length and amino acid composition (Souer et al., 1996; Aida et al., 1997). The NAC transcription factor family has been systematically identified or annotated in many plants including rice, which contains 151 members (Nuruzzaman et al., 2010). NAC genes have been implicated in organ development, secondary wall synthesis, senescence, iron homeostasis, pathogen defenses, and abiotic stress responses (Takada et al., 2001; Guo and Gan, 2006; Kim et al., 2006; Uauy et al., 2006; Zhong et al., 2006; Puranik et al., 2012). The consensus NAC recognition site (NACRS) CGT(G/A) and core DNA binding sequence (CDBS) CACG were first identified in the promoter of *EARLY RESPONSIVE TO DEHYDRATION 1* (*ERD1*) in *Arabidopsis* (Tran et al., 2004). The recognition sequence for NACs might be conserved in plants, since quite a few NACs can bind to this NACRS (Jensen et al., 2010; Sakuraba et al., 2015; Bhattacharjee et al., 2017; Fan et al., 2018). Meanwhile, several stress-related *cis*-elements such as ABRE (ABA responsive element), DRE (dehydration responsive element), salicylic acid responsive element, and jasmonic acid responsive element were also identified in the promoters of many stress-responsive NAC genes (Nakashima et al., 2012). These stress-related *cis*-elements are involved in the regulation of NAC genes under stress conditions.

Various stress-responsive NAC genes have been explored for engineering plants with improved drought resistance (Hu and Xiong, 2014). *ANAC019*, *ANAC055*, and *ANAC072*, which were induced by drought, salt, and ABA, conferred drought tolerance in transgenic *Arabidopsis* (Tran et al., 2004). *SNAC1*-OE rice exhibited better performance under drought and salt stress conditions at the vegetative stage, and the transgenic plants exhibited greater seed production under drought stress conditions at the reproductive stage (Hu et al., 2006). Genotyping of the *SNAC1* promoter in rice germplasm revealed four haplotypes, and the C1 haplotype confers stronger gene induction and showed better drought resistance than other haplotypes under field drought conditions (Songyikhangsuthor et al., 2014). *SNAC2*, a homolog of *SNAC1*, is responsive to various stresses and *SNAC2* overexpressing rice exhibited increased cold tolerance and ABA sensitivity (Hu et al., 2008). Overexpression of *SNAC3*, which is also responsive to diverse stresses, confers increased resistance to both heat and drought stress, mainly through regulating genes for detoxification of reactive oxygen species (ROS) in rice (Fang et al., 2015). Overexpression of *OsNAC10* and *OsNAC5*, which were driven by a root-specific promoter RCc3, enlarged the root diameter in rice, and therefore conferred increased drought resistance and produced more grains under field drought conditions (Jeong et al., 2010, 2013). Overexpression of some NAC genes from one species in another species may also improve drought tolerance. For example, overexpression of *SNAC1* in wheat (Saad et al., 2013) and cotton (Liu et al., 2014) resulted in increased drought tolerance. A rose NAC gene *RhNAC3*, which was induced by dehydration and ABA, confers drought tolerance in transgenic Arabidopsis through osmotic adjustment regulation (Jiang et al., 2014).

Despite numerous reports on the identification and functional analysis of NAC genes, very few NACs have been thoroughly characterized for their direct target genes. Previously, we examined the differentially expressed genes in transgenic rice overexpressing *SNAC1* by microarray analysis (Hu et al., 2006), and two genes, *OsSRO1c* and *OsPP18*, were confirmed to be directly regulated by SNAC1 (You et al., 2013, 2014b). However, because of the technical limitation of the microarray, a complete scenario of the genes directly regulated by SNAC1 remains unclear. In this study, we performed Chromatin immunoprecipitation sequencing (ChIP-Seq) and RNA-Seq to identify SNAC1 target genes at a genome-wide scale. We analyzed the ChIP-Seq and RNA-Seq profiles of *SNAC1*-OE rice and wild-type (WT) under normal and drought stress conditions. We identified 93 SNAC1-targeted genes related to drought resistance. Most of these genes are involved in transcriptional regulation, response to water loss, and other stress processes. We found that SNAC1 can bind to the *OsbZIP23* promoter, a key ABA signaling regulator (Xiang et al., 2008; Zong et al., 2016). Characterization of the SNAC1-targeted genes will provide insights into the molecular mechanism of drought response and new candidates for engineering drought resistance.

## Materials and Methods

### Plant Materials and Growth Conditions

The rice variety Zhonghua 11 (ZH11) and Nipponbare were used in this research. ZH11 was the transgenic receptor and used as the WT control for stress treatments, RNA-Seq, and ChIP-Seq. The full *SNAC1* coding sequence (LOC_Os03g60080) and the *OsbZIP23* promoter sequence (LOC_Os07g15770) were amplified from the total Nipponbare cDNA. The amplified *SNAC1* coding sequence was cloned into pCAMBIA1301U which was driven by a maize ubiquitin promoter with the primer set SNAC1-1301F/R (Supplementary Table S1). The construct was transformed into ZH11 by *Agrobacterium* (EH105)-mediated transformation (Hiei and Komari, 2008). The transgenic and WT seeds were sprouted on half-strength MS medium in the dark for 2–3 days at 28^∘^C, and then 4–5 days in the greenhouse. The seedlings were then moved to pots or a paddy field with sand/paddy (1:3) soil under natural conditions at Wuhan, China.

### Drought Stress Treatment and ABA Treatment at the Seedling Stage

After germination, the 1-week-old seedlings that were of uniform growth status were moved to pots filled with sandy soil. The three independent transgenic lines (OE-14, OE-19, and OE-25) and ZH11 control plants were grown in a half-and-half manner (15 plants each) in the pots. At the 4-leaf stage, the seedlings were subjected to drought stress treatment by stopping water for 10 days until the relative soil water content reached 8–10% and kept for 2–3 days until the leaves were dehydrated and turned yellow. Then, the seedlings were recovered with sufficient water for 5–7 days. The experiment was repeated three times independently, and the final survival rate was determined as the average percentage of recovered seedlings of the total planted of the three replicates.

For ABA sensitivity test, the germinated seeds of the same materials used in the drought treatment were moved to half-strength MS medium containing 3 μmol/L ABA or equal volume of alcohol (as normal condition) and grown for 10 days. Then, the phenotype was recorded and the seedling shoot lengths were measured. For ABA treatment, 100 μmol/L ABA was sprayed to 4-leaf stage seedlings of OE-19, OE-25, and ZH11. After 1 h, the seedlings were harvested and used for RNA extraction.

### Dual Luciferase Assay in Rice Protoplasts

The amplified *SNAC1* full or partial coding sequences were cloned into yeast GAL4 binging domain vectors (GAL4BD) as effectors. Primers used for cloning are listed in Supplementary Table S1. 35S-GAL4-fLUC was used as a reporter to identify the transcription regulation activity of the effectors. AtUbi-rLUC, a Renilla luciferase (rLUC) gene driven by the *Arabidopsis thaliana UBIQUITIN3* promoter was used as an internal control (Hao et al., 2011). All the constructs were extracted by Qiagen Plasmid Midi Kit and dissolved with ddH_2_O to a final concentration of 1 μg/μl. The effectors, reporters, and the internal control were co-transformed into the rice protoplasts in a ratio of 6:6:1 according to the reported method (Xie and Yang, 2013). After culturing 12 h in the dark at 25^∘^C, the protoplasts were collected and luciferase activities measured using a Dual-Luciferase Reporter Assay System Kit (Promega) and a Tecan Infinite M200 Microplate Reader. The ratio of fLUC to rLUC activity represents the transcriptional activity of the effectors.

### RNA Extraction and Real-Time PCR Analysis

The total RNAs were extracted from the leaves of *SNAC1*-OE lines (OE-19 and OE-25) and ZH11 plants using TRIzol reagent (Invitrogen^TM^) according to the manufacturer’s instructions. Then the RNA (3 μg) was reverse-transcribed using the EasyScript One-Step gDNA Removal and cDNA Synthesis Kit (TransGen^TM^). Subsequently, the reverse transcription product was diluted ten times with ddH_2_O and 2 μl was taken as the template for real-time quantitative PCR (q-PCR). q-PCR was performed using a QuantStudio 6 Flex Real-Time PCR System (Applied Biosystems) analyzer and each reaction contained 5 μl 2 × PowerUp^TM^SYBR Green Master Mix (Applied Biosystems^TM^), 0.2 μM of each gene-specific primer (primer sequences are listed in Supplementary Table S1). The gene expression level was calculated using the 2^–ΔΔCT^ method based on three independent replicates and a rice *Ubiquitin* gene (LOC_Os03g13170) was used as an internal control.

### RNA-Seq and Transcriptome Profiling Analysis

The total RNAs were extracted from the leaves of *SNAC1*-OE line OE-19 and ZH11 plants at the 4-leaf stage with three independent replicates. The RNA libraries were sequenced by Novogene (Tianjin, China) with Illumina^®^ Hiseq X Ten, and we used the BMKCloud cloud server^1^ to analyze the raw data which was mapped to the rice MSU 7.0 reference genome^2^. Gene expression levels were measured by the FPKM (Fragments Per Kilobase of exon model per Million mapped reads) method (Florea et al., 2013). The criterion of differentially expressed genes (DEGs) was the log_2_FC ≥ 1 as well as the false discovery rate (FDR) < 0.05.

### Chromatin Immunoprecipitation (ChIP) Assay and ChIP Sequencing

The ChIP assay was performed according to Bowler et al. (2004) with some modifications. Briefly, 3 g shoot tissues from 4-leaf stage seedlings were cross-linked in 1% formaldehyde by vacuum for 30 min. The chromatin was extracted on ice as described by Bowler and sheared to 200–500 bp fragments for ChIP-Seq or 200–1000 bps fragments for ChIP-PCR by sonication. Subsequently, the DNA fragments were immunoprecipitated by anti-SNAC1 polyclonal antibody from rabbits prepared by ABclonal (Wuhan, China). The combined DNAs were eluted, purified, and dissolved in ddH_2_O. Primers used in ChIP-qPCR are listed in Supplementary Table S1.

The materials used for ChIP-Seq were the same as with RNA-Seq (also with three independent replicates), notably, three kinds of samples were analyzed by ChIP-Seq, except for OE-19 plants under drought stress conditions. The ChIP libraries were sequenced by Novogene (Tianjin, China) and preliminary analysis of the raw data was performed on the BMKCloud cloud server as well. We compared the clean data to the rice MSU 7.0 reference genome using bowtie2 (Langmead et al., 2009). The MACS2 software (Zhang et al., 2008) was used to find the potential interaction regions between DNA and proteins. The conserved motifs that SNAC1 can bind to were analyzed by MEME-ChIP (Machanick and Bailey, 2011).

### Statistical Analysis

All experiments were repeated independently three times. The Student’s *t*-test and the Least Significant Difference were calculated using SPSS^3^.

### Accession Numbers

The RNA-Seq and ChIP-Seq data in this article were deposited in the Gene Expression Omnibus under accession number GSE128495.

## Results

### SNAC1 Transcriptional Activation Assay

**FIGURE 1:**
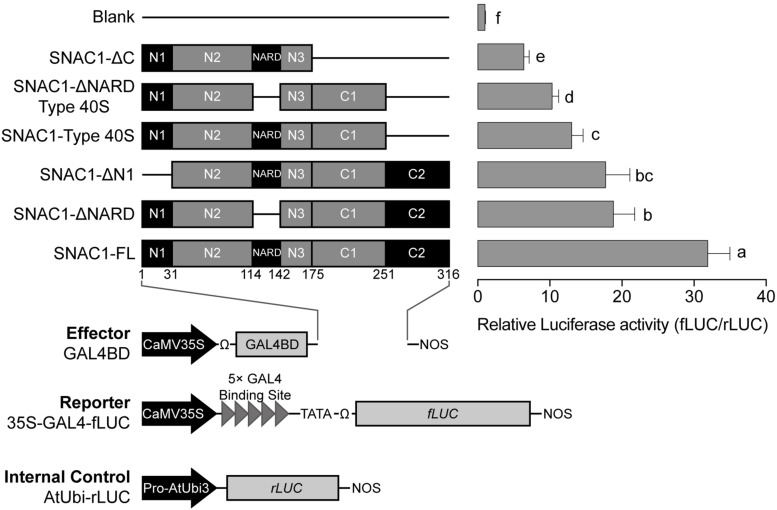
Full (*SNAC1-FL*) or partial (*SNAC1*-ΔC, *SNAC1*-ΔNARD Type 40S, *SNAC1*-Type 40S, *SNAC1*-ΔN1, *SNAC1*-ΔNARD) length of the SNAC1 fragments fused with the firefly luciferase reporter were tested for transactivation activity. The deletions are shown in lines. The left panel in the figure shows the structure of each vector, and the right panel shows the fLUC/rLUC activity ratio in the protoplasts. The ratio obtained from the transfection of the blank vectors (Blank) was set arbitrarily as 1. Error bar indicates the standard deviation (SD) of three independent replicates. Different letter(s) beside each column indicates the significant difference calculated via the Least Significant Difference (LSD) method (α = 0.05).

In a previous study, the *SNAC1* cDNA isolated from a cDNA library for overexpression (Hu et al., 2006) actually encoded a protein with an incomplete C-terminus, which lacked 251–316 AA of the complete reference sequence (SNAC1-Type40S, Figure 1). Even though the SNAC1-Type40S is incomplete, it showed strong transactivation activity in yeast, and overexpression of this form in rice resulted in a significant increase in drought resistance (Hu et al., 2006). We tested the transactivation activity of the full-length SNAC1 (SNAC1-FL) using a dual luciferase assay and found that SNAC1-FL had stronger transactivation activity than SNAC1-Type40S in rice protoplasts (Figure 1). A NAC repression domain (NARD), which has been found in other NACs such as GmNAC20 (Hao et al., 2010), was also identified in the middle of the SNAC1 protein sequence (the 114–142 AA, Figure 1). We then tested the effect of the NARD on transactivation activity in rice protoplasts. To our surprise, the truncated SNAC1 without the NARD (SNAC1-ΔNARD) showed weaker activity than the SNAC1-FL (Figure 1), suggesting that the NARD in SNAC1 may not act as a repression domain. Besides, deletion of the first 31 AAs of SNAC1-FL reduced its activity to a level similar to SNAC1-ΔNARD, indicating that the SNAC1 N-terminus accounted for part of the transcriptional activity. In addition, the incomplete version with deletion of the C-terminal region (lacking the 176–316 AA, SNAC1-ΔC) showed the least activity among the vectors tested (Figure 1), suggesting that the SNAC1 C-terminus was indispensable for maintaining the activity of SNAC1. These results indicated that the intact SNAC1 coding sequence is essential for its full activity.

### Overexpression of *SNAC1-FL* Resulted in Improved Drought Resistance and ABA Sensitivity

**FIGURE 2:**
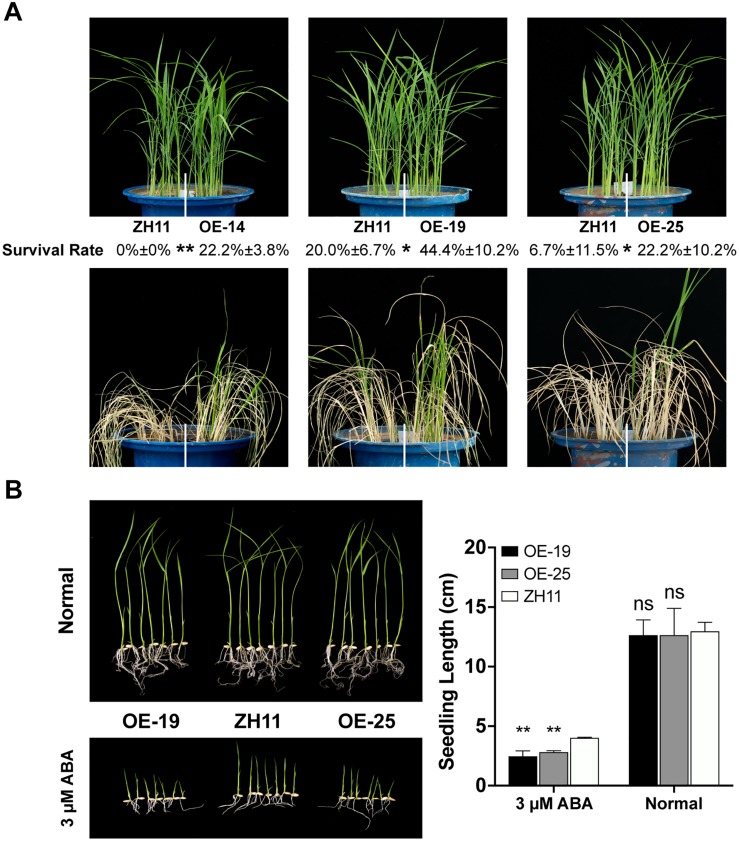
*SNAC1*-OE plant phenotypes under drought stress and ABA treatment. **(A)** Drought resistance testing of *SNAC1*-OE lines. The survival rates of the *SNAC1*-OE and ZH11 plants after recovery are expressed as mean ± SD of three independent replicates. **(B)** Performance of *SNAC1*-OE (OE-19 and OE-25) and ZH11 in half-strength MS medium containing 0 (normal) or 3 μmol/L ABA. The measurement of seedling length after 10 days’ treatment is shown by the histogram. Error bar represents the SD of three independent replicates. Asterisk(s) in the figure represents the significant difference (OE versus ZH11) determined by the paired Student’s *t*-test (^∗∗^*p* < 0.01, ^*^0.01 ≤ *p* < 0.05, ns: *p* ≥ 0.05).

Since overexpression of *SNAC1-Type40S* in rice variety Nipponbare resulted in significantly increased drought resistance (Hu et al., 2006), and SNAC1-FL showed stronger transactivation activity, we assumed that SNAC1-FL may also confer drought resistance. To confirm this, we overexpressed *SNAC1-FL* in rice ZH11, a genotype showing more drought tolerance than Nipponbare (Guo et al., 2018), and the independent transgenic lines were tested for drought resistance and ABA sensitivity. For drought testing at the seedling stage, germinated seeds of three *SNAC1-FL* overexpression (*SNAC1*-OE) lines (OE-14, OE-19, and OE-25) and ZH11 control were transplanted into blue barrels filled with a mixture of sand/paddy (1:3) soil. The *SNAC1*-OE lines and ZH11 seedlings were subjected to drought stress treatment at the 4-leaf stage. The water content of the soil was maintained at 8–10% for 2–3 days, and then recovered with saturated watering for 7 days. All three *SNAC1*-OE lines showed significantly increased drought resistance compared to ZH11 in terms of survival rate after recovery (Figure 2A). We then tested the growth performance of two OE lines (OE-19 and OE-25) on ABA (3 μmol/L) containing MS medium. The growth of both OE lines was significantly slower than that of ZH11, while in normal medium without ABA no difference was observed between the OE lines and ZH11 (Figure 2B). These results suggested that the full length SNAC1 also confers drought resistance and ABA sensitivity, with similar performance to the *SNAC1-Type40S* overexpression lines (Hu et al., 2006).

### Transcriptome Analyses of *SNAC1-FL* Overexpression Lines

**FIGURE 3:**
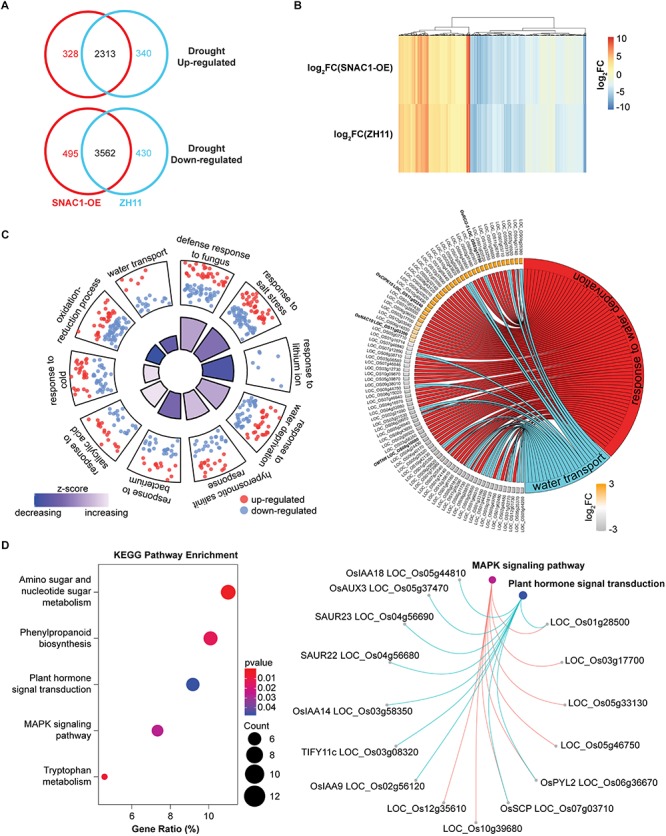
Transcriptome profiling of *SNAC1*-OE (OE-19) plants. **(A)** Numbers of drought-responsive DEGs in the ZH11 and OE-19 plants. **(B)** Heatmap showing the expression pattern of the 980 SRDGs in the OE-19 and ZH11 plants in response to drought. **(C)** Top 10 significantly enriched GO biological processes of the SRDGs. Red and blue dots indicate up-regulated DEGs and down-regulated DEGs enriched in the term respectively, and a *z*-score indicated in the inner quadrangle represents the overall log_2_FC trends of the DEGs within the enriched term. Proportion of the inner quadrangle is in accordance with the –log_10_(*p*-value), demonstrating the significance of the enrichment. Genes involve in the GO term “response to water deprivation” and “water transport” are shown in the circular plot (right). The log_2_FC of each gene in OE-19 plants in response to the drought stress condition is marked by a colored rectangle. Genes mentioned in the article are highlighted in bold. **(D)** KEGG pathway enrichment of the SRDGs. Genes involved in “MAPK signaling pathway” and “Plant hormone signal transduction” are exhibited with a circular network plot (right). All DEGs were determined by the threshold of log_2_FC > 1 and *p*-adjust < 0.05 with three independent replicates.

Since *SNAC1* confers drought resistance as a transcription factor, identification of the genes regulated by SNAC1 under drought stress conditions would help reveal the molecular mechanisms of drought resistance. For this purpose, we compared the genome-wide transcript profiles of *SNAC1*-OE line OE-19 and ZH11 at the seedling stage under normal and moderate drought stress conditions by using RNA-Seq. The FPKM method (Florea et al., 2013) was used to evaluate the relative gene expression level, and a threshold of twofold change (log_2_FC > 1) between samples with FDR between repeats less than 0.05 was used to claim differentially expressed genes (DEGs). Using these criteria, a total of 6698 and 6645 MSU 7.0 annotated DEGs were called in OE-19 and ZH11, respectively, in response to drought stress treatment. In the OE-19 line, 2641 and 4057 genes were up and down-regulated, respectively, while in the ZH11, 2653 and 3992 genes were up and down-regulated, respectively (Figure 3A). To identify drought-regulated genes that were also regulated by SNAC1, we selected 980 candidate genes, whose log_2_FC value in each sample varied more than 0.5, from all 5874 overlapping DEGs. These 980 genes were considered as the SNAC1-regulated genes under drought stress conditions (SRGDs, Figure 3B and Supplementary Table S2). To evaluate the reliability of the RNA-Seq data, 15 DEGs were randomly chosen for qPCR analyses, and the results showed that the relative expression levels of these genes examined by q-PCR and RNA-Seq were highly correlated (Supplementary Figure S1).

To further elucidate the putative function of the SRGDs, we performed a gene ontology (GO) enrichment analysis on these 980 genes. As is shown in Figure 3C, several biological processes in relation to stress response were enriched, whereas most enriched terms contained more down-regulated genes than up-regulated genes. Although we characterized SNAC1 as a transcription activator, the result was still acceptable since there were more drought down-regulated genes than up-regulated genes in both group (3562 versus 2313, Figure 3A). We then focused on two GO terms related to water regulation, and among the 85 enriched genes, several reported drought responsive genes were observed. For example, drought resistance contributing genes *OsNAC10* (LOC_OS11g03300) (Jeong et al., 2010), *OsCIPK15* (LOC_OS11g02240) (Xiang et al., 2007), and *OsRCI2-5* (LOC_OS03g17790) (Li et al., 2014) were found in the up-regulated genes and drought resistance negative-regulating NAC transcription factor gene *OMTN6* (LOC_OS08g10080) (Fang et al., 2014) was involved in the down regulated genes. The transcriptional altering of such crucial drought responsive genes in the *SNAC1*-OE line indicated the status of SNAC1 in drought response regulation.

To find a possible biological link between the DEGs and the increased drought resistance and ABA sensitivity in the *SNAC1*-OE rice, KEGG (Kyoto Encyclopedia of Genes and Genomes) annotation and enrichment were conducted for the SRGDs. The results indicated that the SRGDs were enriched in several metabolism pathways, which may contribute to the maintenance of cell function under drought stress conditions (Figure 3D). Notably, many auxin responsive genes were enriched in “Plant hormone signaling transduction”, and some of these *OsIAA* genes were also reported as ABA responsive genes and participated in drought response and the crosstalk between ABA and IAA signaling (Song et al., 2009; Zhao et al., 2015; Wang M. et al., 2018). These results suggested that SNAC1 may play a role in the regulation of ABA-IAA signal interaction, and partially explained the ABA hyper-sensitivity of *SNAC1*-OE seedlings.

### Genome-Wide Identification of SNAC1-Binding Sequences

**FIGURE 4:**
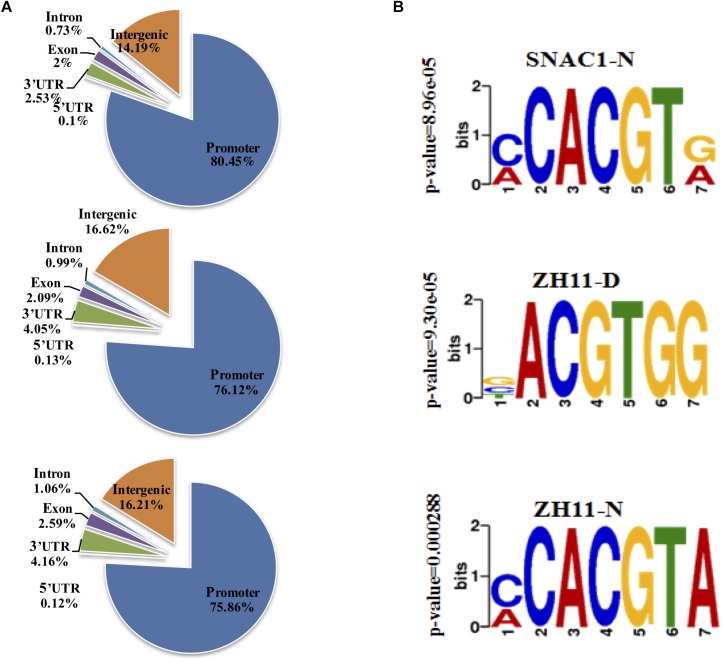
Genome-wide distribution of SNAC1 binding sites in the rice genome identified by ChIP-Seq. **(A)** Distribution of SNAC1 binding sites within rice genic regions in SNAC1-N, ZH11-D, and ZH11-N. **(B)** Motif enrichment analysis of SNAC1-bound sites. All binding profiles were determined with three independent replicates.

Since SNAC1 is a typical transcription factor with a DNA binding domain, we tried to identify all the genes with their promoters potentially bound by SNAC1. For this purpose, a SNAC1-specific antibody was generated to conduct ChIP-Seq using leaf samples from OE-19 and ZH11 plants at the seedling stage. The summited peaks resulting from ChIP-Seq were annotated by correlated genes and proximal genomic features. We found that more than 75% of the peaks are located in the promoters (−2000 to 0 bp region in relation to the TSS, Figure 4A), which agrees with the role of SNAC1 as a DNA-binding transcription factor. This result also reflected the high quality of the ChIP-Seq data for further identification of SNAC1-targeted genes.

In Arabidopsis, the NAC recognition sequence (NACRS) with CACG as a core motif has been identified in the promoters of several NAC-targeted genes (Fujita et al., 2004), and such NACRS could be bound by SNAC1 (Hu et al., 2006). We analyzed all the promoter-located peak sequences from the ChIP-Seq using MEME-ChIP to identify the enriched motif, and detected the three most enriched motifs, CACGT, CACGTA, and ACGTGG in SNAC1-N (OE-19 under normal growth conditions), ZH11-N (ZH11 under normal growth conditions), and ZH11-D (ZH11 under drought stress conditions), respectively (Figure 4B). Obviously, both the CACGT and CACGTA motifs contain CACG, suggesting that the NACRS may be conserved in plants. Although the most enriched motif in ZH11-D does not contain a complete CACG, the sequence is highly similar to the motifs under normal growth conditions. Interestingly, all three enriched motifs contain ACGT, which is the core motif of ABA responsive element (ABRE). It is possible that SNAC1 prefers to bind to the CACG-containing sequences under normal growth conditions, but under drought stress conditions, it prefers to bind the ACGTGG motif, which is more closely related to ABA response and thus regulate ABA-responsive genes.

### Identification of SNAC1-Targeted Genes Under Drought Stress Conditions

**FIGURE 5:**
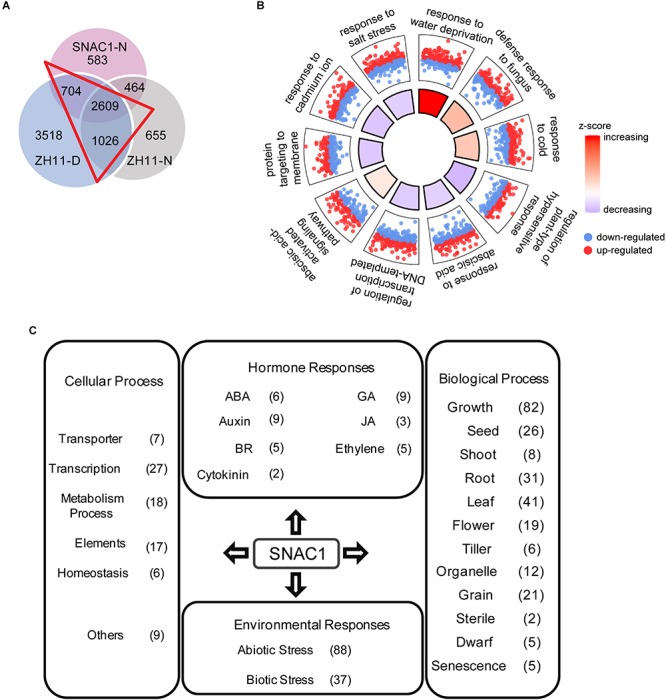
ChIP-Seq analysis of putative SNAC1-binding sites (genes). **(A)** Overlapping numbers of SNAC1-binding genes among the three samples: SNAC1-N, ZH11-D, and ZH11-N. The numbers in red triangle collectively represent the putative SNAC1-binding genes involved in drought response (SNAC1BGDs). **(B)** GO enrichment analysis on SNAC1BGDs. Top 10 significantly enriched GO terms in the biological processes category are shown. **(C)** SNAC1BGDs with known functions in various cellular processes and responses.

According to the genomic locations of the ChIP-Seq peaks, a total of 5064, 7857, and 4754 genes with peak(s) in the promoter region were identified in SNAC1-N, ZH11-D, and ZH11-N, respectively, and 2609 genes were present in all three samples. To evaluate the reliability of the ChIP-Seq data, we randomly chose six SNAC1-bound genes and two non-SNAC1-bound genes for ChIP-qPCR analysis, and the result was consistent with the ChIP-Seq data (Supplementary Figure S2). In this study, we were more interested in the SNAC1-bound genes under drought stress conditions. Therefore, we chose the overlapping genes between SNAC1-N and ZH11-D, plus the overlapping genes between ZH11-D and ZH11-N, which resulted in 4339 genes, and these genes were defined as SNAC1-bound genes involved in drought stress (SNAC1BGD) (Figure 5A).

Gene ontology analysis of the SNAC1BGD genes revealed significant enrichment in transcriptional regulation, response to diverse stresses including water deficit, salinity, cold, and ABA (Figure 5B). Among these genes, 504 were reported and could be classified into different categories according to their putative functions, and the genes involved in stress responses and developmental regulation were obviously more numerous than the other categories (Figure 5C and Supplementary Table S3). Notably, several reported genes functioning in stress tolerance, such as *OsbZIP72* (Lu et al., 2009), *COLD1* (Ma et al., 2015), *OsbZIP23* (Zong et al., 2016), and *DHS* (Wang Z. et al., 2018), were in the SNAC1BGD.

**FIGURE 6:**
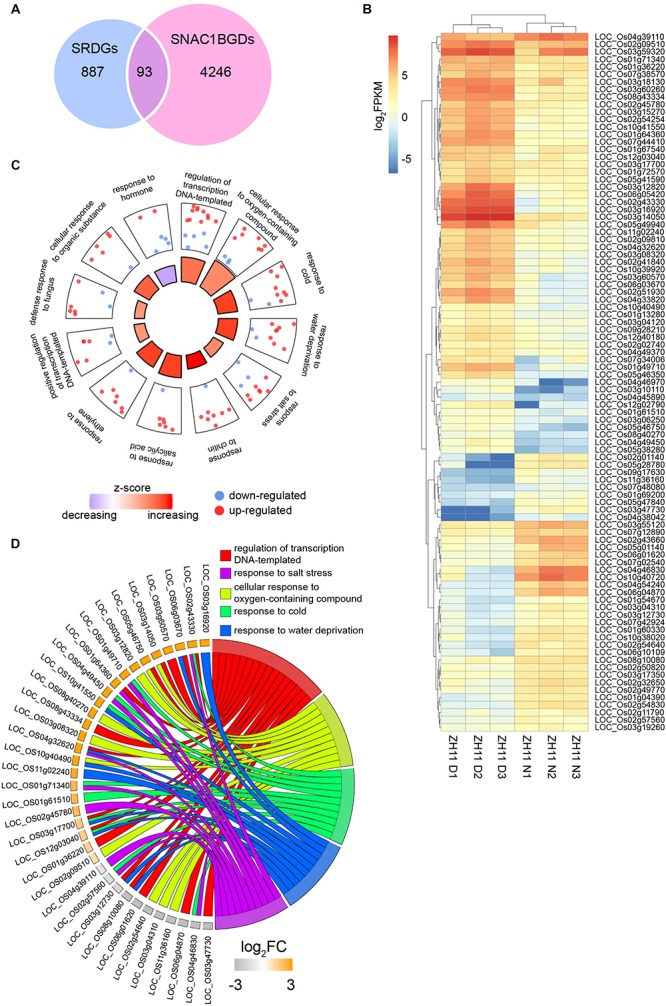
Characterization of the 93 SNAC1TGDs. **(A)** Overlapped number of genes between the 4339 SNAC1BGDs from ChIP-Seq analysis and the 980 SRDGs from RNA-Seq analysis. **(B)** Expression patterns of the SNAC1TGDs in ZH11 in response to drought stress. **(C)** First 12 GO enrichments in the biological process category of the SNAC1TGDs. **(D)** Circular plot exhibiting genes involved in stress response related GO terms enriched in **(C)**.

Next, we combined the 980 SRDGs from the RNA-Seq analysis with the 4339 SNAC1BGD genes, and found that only 93 genes were overlapping between these two datasets. These 93 genes represent the genes that are not only directly bound by SNAC1 in their promoters, but are also regulated by SNAC1 under drought stress conditions, and therefore defined as SNAC1 target genes involved in drought response (SNAC1TGDs) (Figure 6A and Supplementary Table S4). Among these 93 genes, 55 were up-regulated and 38 were down-regulated by the drought stress treatment according to the ZH11 RNA-Seq results (Figure 6B). GO analysis of the SNAC1TGDs revealed significant enrichment in several abiotic stress related biological processes such as “response to water deprivation”, “response to oxygen-containing compound”, and “response to salt/cold stress” (Figure 6C and Supplementary Table S5). Moreover, 11 out of the top 12 enriched GO terms involved a greater number of up-regulated genes than down-regulated genes, and showed strong trends of up-regulation (with *z*-scores > 1), suggesting the transcriptional activation character of SNAC1. Of special note, although most of the GO enriched genes have not been experimentally characterized, a previously identified SNAC1 targeted gene *OsSRO1c* (LOC_Os03g12820) was involved in the “cellular response to oxygen-containing compound” GO term of SNAC1TGD, and up-regulated by drought stress conditions (Figure 6D). It has been reported that elevated expression of *OsSRO1c* conferred enhanced drought resistance by conducting the accumulation of cellular H_2_O_2_ (You et al., 2014a). These results emphasized the importance of SNAC1 in H_2_O_2_ mediated drought response regulation.

### SNAC1 Positively Regulates ABA Pathway Genes

**FIGURE 7:**
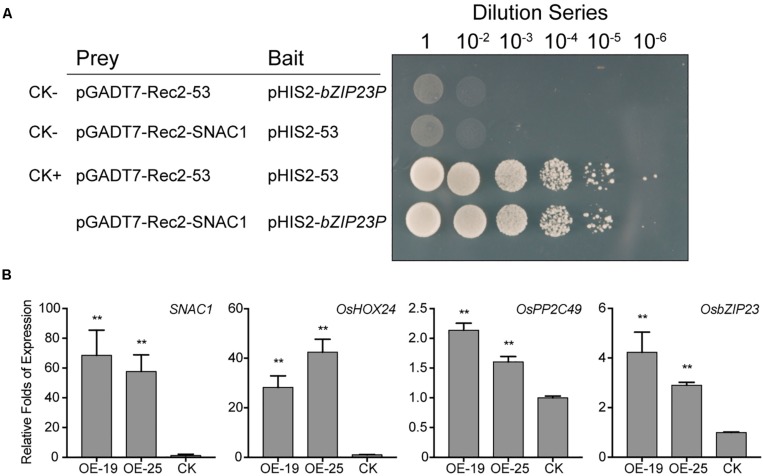
SNAC1 function may partially depend on the ABA signaling pathway. **(A)** Yeast-one-hybrid assays indicate the direct interaction between SNAC1 and the *OsbZIP23* promoter. CK–, negative control; CK+, positive control. **(B)** The expression level of *SNAC1*, *OsPP2C49, OsbZIP23*, and *OsHOX24* in the *SNAC1*-OE lines OE-19 and OE-25 and ZH11 treated with 100 μmol/L ABA for 1 h at the seedling stage. Error bar indicates the SD of three independent replicates. Asterisks indicate the significant difference (OE versus ZH11) in the ddCt values used to calculate the relative expression levels (^∗∗^*p* < 0.01, by Student’s *t*-test).

The *SNAC1*-OE transgenic rice plants were hypersensitive to ABA (Figure 2B), and among the SNAC1BGD, there were several genes related to ABA signaling such as *OsPP2C49* (Zong et al., 2016), *OsNCED3* (Bang et al., 2013), *OsbZIP23* (Xiang et al., 2008; Zong et al., 2016), and *OsHOX24* (Bhattacharjee et al., 2016). Yeast-one-hybrid assay showed that SNAC1 was able to bind to the *OsbZIP23* promoter (Figure 7A). We further examined whether SNAC1 was involved in the regulation of ABA signaling related genes. qPCR assay on the 100 μmol/L ABA treated 4-leaf stage seedlings of the two *SNAC1*-OE lines (OE-19 and OE-25) and ZH11 revealed that the relative expression levels of *OsPP2C49*, *OsHOX24*, and *OsbZIP23* were significantly greater in both OE lines than that in the ZH11 control (Figure 7B). The result suggested that SNAC1 may positively regulate the expression of these genes in response to ABA.

## Discussion

The NAC family are plant specific transcription factors playing important roles in the regulation of plant development and stress responses. However, limited studies have been reported for genome-wide identification of genes regulated by specific NAC proteins. SNAC1 has been reported as a key drought resistance regulator in rice (Hu et al., 2006), but the mechanism remains largely unclear. By combining the ChIP-Seq and RNA-Seq techniques, we obtained profiles for both the SNAC1-bound genes and the genes regulated by SNAC1. These data provide information to further elucidate the molecular mechanisms of SNAC1 in regulating drought resistance.

### SNAC1 Regulates Numerous Genes Related to Stress Responses and Development

Although *SNAC1* is strongly induced by drought stress, it has a relative high expression level under normal growth conditions (Hu et al., 2006). Since *SNAC1*-OE lines showed increased drought resistance, we were more interested in the genes that are regulated by SNAC1 under drought stress conditions. Among these genes, more than 30% have been annotated with putative functions involved in response to diverse stresses or phytohormones. Notably, there are several enriched GO categories related to stress tolerance. For example, *OsJAZ7* and *OsJAZ12*, which belong to the OsTIFY family, are enriched in the SNAC1BGD and are annotated in “responses to abiotic stimulus” (GO:0009628). Both *OsJAZ7* and *OsJAZ12* contain the Jas motif and were actually induced by JA, suggesting their functions in promoting plant defense against abiotic stresses (Ye et al., 2009; Verma et al., 2016). *OsDREB1B* and *OsPYL*, well-known genes related to abiotic stress and ABA response, were detected in “response to stress” (GO:0006950). *OsDREB1B* was induced not only by low temperature, but also by drought and mechanical stress, and overexpression of *OsDREB1B* in tobacco showed improved water retention and stress tolerance (Dubouzet et al., 2003; Gutha and Reddy, 2008; Figueiredo et al., 2012). *OsPYL*, a rice homolog of *PYL* that encodes an ABA receptor in Arabidopsis, has been considered as an important gene for regulating ABA-dependent gene expression in rice (Kim et al., 2012, 2014). The improved drought resistance of the *SNAC1*-OE rice might be the result of the integrated effect of these genes.

Despite the genome-wide identification of SNAC1-regulated genes, we aimed to discover the genes that are directly regulated by SNAC1 under drought stress conditions. The results suggest that, among the 4339 SNAC1BGD genes, there are also numerous genes related to stress responses and phytohormone signaling. Although some of the SNAC1BGD genes were actually regulated by SNAC1 at the transcript level, some SNAC1BGD genes showed no significant changes in transcript level under drought stress conditions. We assume that other transcription factors may be required to jointly regulate the expression of these genes. We noticed that some drought resistance-related regulatory genes were up-regulated by SNAC1, such as *ONAC095* (Huang et al., 2016), *ARAG1* (Zhao et al., 2010), *OsbZIP23* (Zong et al., 2016), *OsSKIPa* (Hou et al., 2009), *OsMYB55* (El-Kereamy et al., 2012), and *OsMYC2* (Cai et al., 2014). This may partially explain that there are numerous genes that are not directly bound by SNAC1 but can also be regulated though SNAC1-targeted transcription factors.

In addition to the stress-related genes, some of the SNAC1BGD genes have been reported or annotated with putative functions in development. For example, OsMADS6 directly targets *FACTOR OF DNA METHYLATION LIKE 1 (OsFDML1)* and controls flower development (Tao et al., 2018). *OsDOS* is down-regulated during natural leaf senescence, panicle development, and pollination, and it delays leaf senescence by partly integrating developmental cues to the JA pathway (Kong et al., 2006). *HOX12* is predominantly expressed in panicles and plays an important role in panicle exertion from the flag leaf sheath in rice (Gao et al., 2016). Therefore, SNAC1 may also function in developmental processes through the regulation of these genes. We generated a *SNAC1*-knockout mutant, and the mutant showed abnormal morphology and defects in spikelet fertility, further confirming that SNAC1 is involved in development regulation.

### SNAC1 Participates in Regulation of ABA-Dependent Pathways

ChIP-Seq results suggest that SNAC1 could bind not only to the NACRS, which was first identified in Arabidopsis (Tran et al., 2004) and confirmed as a NAC-binding site in other species such as *Brassica napus* (Yan et al., 2018) and rice (Sun et al., 2012), but also to the ABRE-like elements especially under drought stress conditions (Figure 4B). The relevance of the NAC transcription factor to ABRE has also been reported in other species. For example, a wheat NAC transcription factor TaNAC47 was reported with ABRE-binding activity in yeast (Zhang et al., 2015). In the *RD29A* promoter, a marker gene for drought-response in Arabidopsis, an ABRE is located between two NACRS and the two types of *cis*-elements can be bound by ABF2/4 and ANAC096, respectively, to jointly regulate ABA-responsive genes involved in dehydration and osmotic stress tolerance (Xu et al., 2013).

The binding of ABRE-like elements by SNAC1 suggested that SNAC1 may regulate drought resistance partially through ABA-dependent pathways. In fact, several important ABA signaling genes such as *OsbZIP23* and *OsPP2C49* were up-regulated in the *SNAC1*-OE rice (Figure 7), and numerous ABA-responsive genes were differentially expressed in the overexpression rice under drought conditions (Supplementary Table S4). Interestingly, our previous study suggested that OsbZIP23, a key transcription factor mediating ABA signaling, could bind to the *SNAC1* promoter and activate its expression (Zong et al., 2016). These results imply that OsbZIP23 and SNAC1 can directly regulate each other to amplify the ABA signaling. Since SNAC1 can regulate ABA signaling, it is not surprising to see the phenotype that the *SNAC1*-OE rice showed increased ABA sensitivity (Figure 2B). Considering the important roles of OsbZIP23 and SNAC1 in drought resistance, and the direct regulation by each other, it is promising to engineer these genes simultaneously using suitable promoters to improve drought resistance in crops.

## Author Contributions

LX conceived the study and designed the experiments. XL designed the experiments, generated the transgenic materials, and performed most of the experiments. YC performed some experiments. SM and JS provided assistance on the ChIP experiments. HH provided advice on the study. XL, HH, and LX wrote the manuscript.

## Conflict of Interest Statement

The authors declare that the research was conducted in the absence of any commercial or financial relationships that could be construed as a potential conflict of interest.

## Footnotes

1 http://www.biocloud.net/

2 http://rice.plantbiology.msu.edu

3 https://www.ibm.com/analytics/spss-statistics-software

## Supplementary Material

FIGURE S1 Correlation analysis between q-PCR and the RNA-Seq data.

FIGURE S2 Enrichment of six SNAC1 bound and two unbound genes examined by ChIP-qPCR with the same samples for ChIP-Seq.

TABLE S1 Primers used in this study.

TABLE S2 Differentially expressed genes identified from RNA-Seq.

TABLE S3 Published genes in the SNAC1BGD data set.

TABLE S4 Detail information for the SNAC1TGD genes.

TABLE S5 Detail information for the GO enrichment of the SNAC1TGD genes.
