# Supplementary figures and images for: Genome-Wide Identification of SNAC1-Targeted Genes Involved in Drought Response in Rice
Source: Front Plant Sci. 2019 Jul 26;10:982. doi: 10.3389/fpls.2019.00982 (PMC6677020; doi:10.3389/fpls.2019.00982)

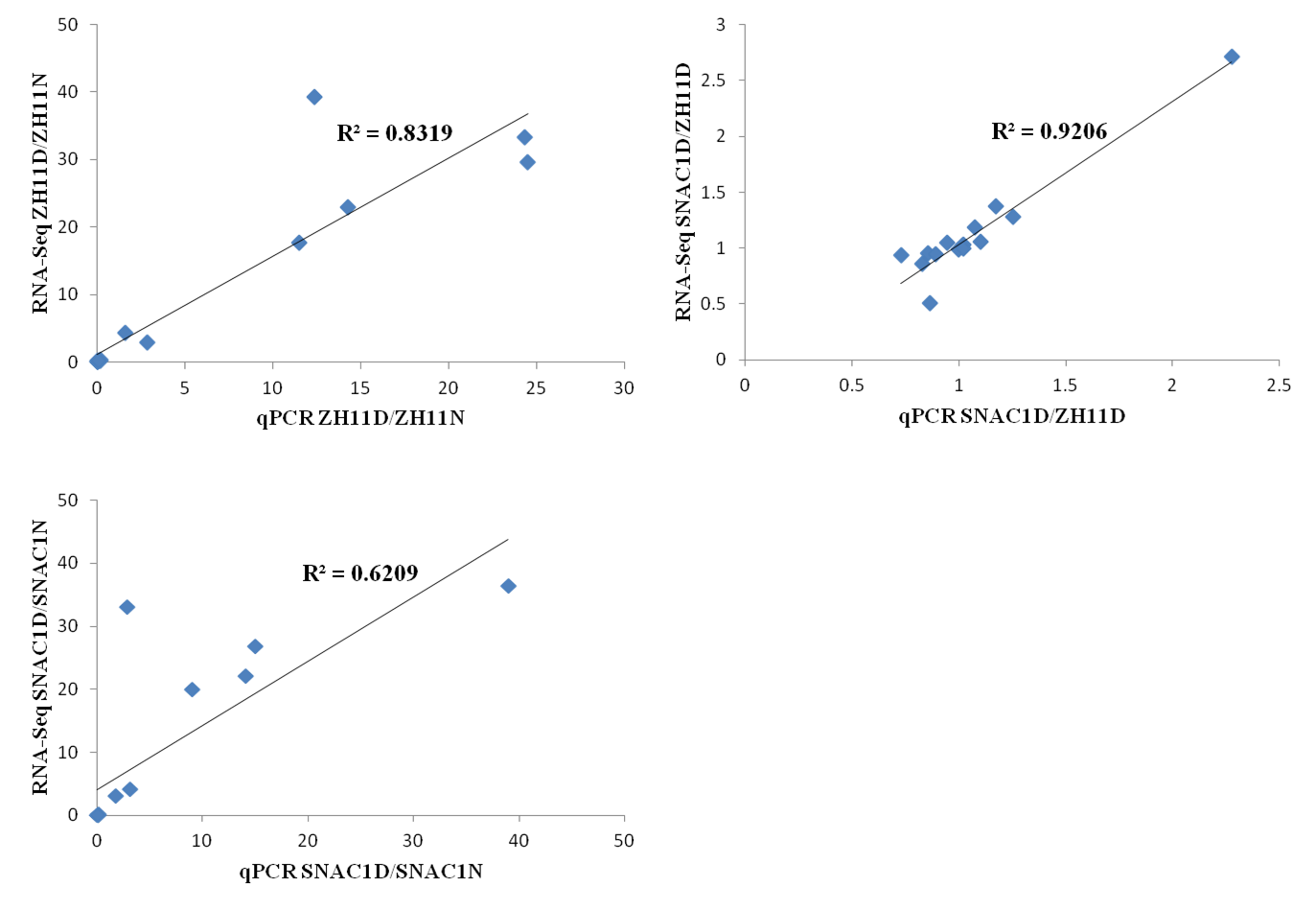

Supplement: FIGURE S1 — Correlation analysis between q-PCR and the RNA-Seq data. [file Image_1.TIF]

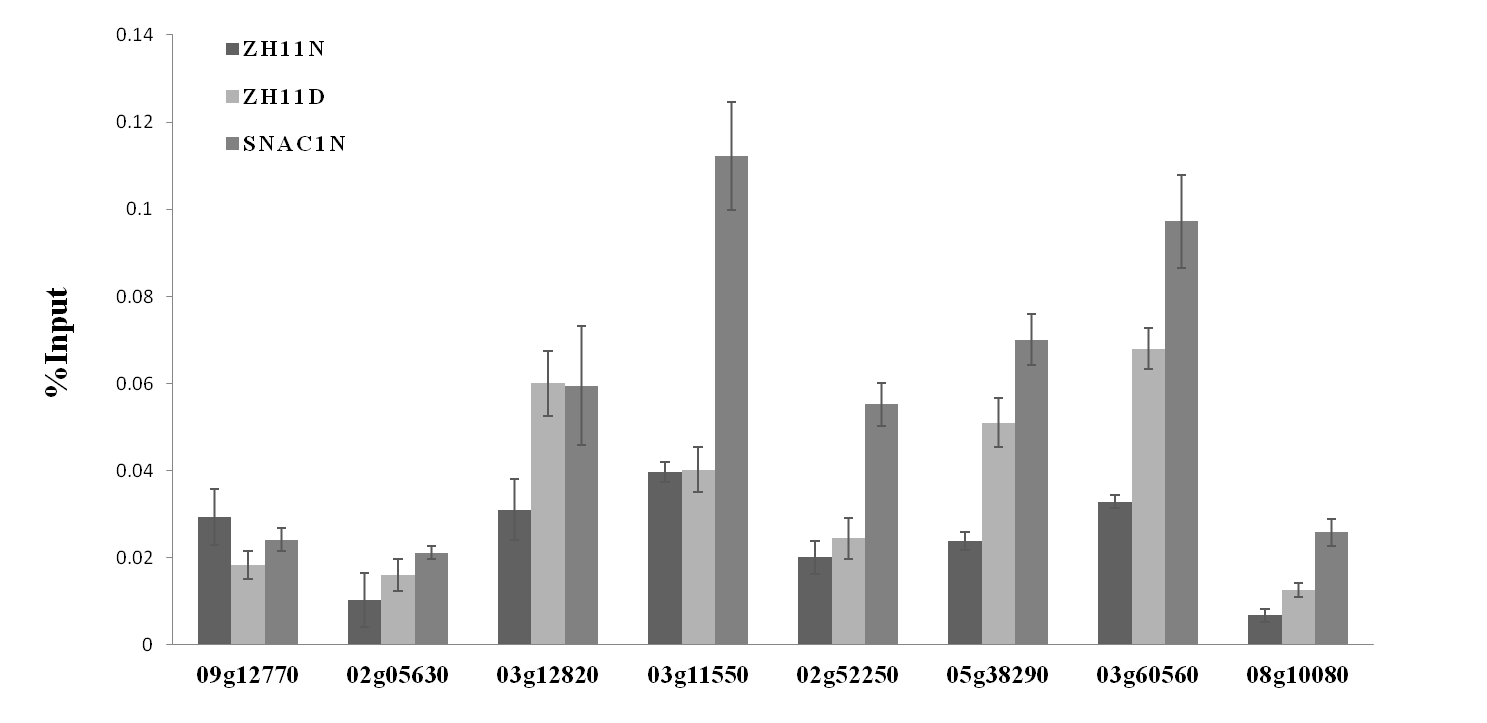

Supplement: FIGURE S2 — Enrichment of six SNAC1 bound and two unbound genes examined by ChIP-qPCR with the same samples for ChIP-Seq. [file Image_2.TIF]
